# Understanding the biochemical impact of leukoreduction on canine pRBC storage: a focus on reactive oxygen species and storage lesions

**DOI:** 10.3389/fvets.2025.1563532

**Published:** 2025-09-04

**Authors:** Sun Woo Shin, Minji Kim, Chaewon Shin, Hyeona Bae, Jinho Park, Dong-In Jung, Kyu-Woan Cho, DoHyeon Yu

**Affiliations:** ^1^College of Veterinary Medicine, Gyeongsang National University, Jinju, Republic of Korea; ^2^College of Veterinary Medicine, Jeonbuk National University, Iksan, Republic of Korea

**Keywords:** leukoreduction, reactive oxygen species, storage lesion, dog, transfusion

## Abstract

Transfusion therapy is vital for both humans and animals, though it poses significant risks, including the development of storage lesions in packed red blood cells (pRBCs). This study examines hematological and biochemical changes during the storage of canine pRBCs, focusing on intraerythrocytic reactive oxygen species (ROS) and the impact of pre-storage leukoreduction. Eleven pRBC units were each divided into two aliquots, resulting in a total of 22 units, eleven leukoreduced (LR-pRBC) and eleven non-leukoreduced (nLR-pRBC), which were analyzed over 42 days. Results showed increased hemolysis, lactic acidosis, and potassium efflux (All, *p* < 0.01), with more severe lesions in nLR-pRBCs due to leukocyte presence. Notably, intraerythrocytic ROS levels increased in both groups (*p* < 0.05), driven by hemoglobin autoxidation (*p* < 0.05), though they decreased in later storage stages due to hemolysis and membrane vesiculation. The study highlights that pre-storage leukoreduction mitigates storage lesions, suggesting its implementation to enhance pRBC storage safety. Further research is necessary to understand the role of antioxidant systems in controlling intraerythrocytic ROS and preventing storage lesions.

## 1 Introduction

Transfusion therapy is essential for both humans and animals, yet it carries the risk of severe complications (1, 2). Recent advancements in transfusion medicine have enhanced the availability and safety of blood products for canine patients. These improvements encompass blood typing, cross-matching, appropriate transfusion methods, dosage optimization, and leukoreduction (3, 4). As the demand for blood products rises, ensuring the safety of hemocomponents becomes increasingly critical. Unfortunately, standards for the storage of canine red blood cells (RBCs) are currently limited and not universally established.

The routine storage period of canine RBC units varies depending on the anticoagulant and preservation solution employed, typically ranging from 35 to 42 days (3, 4). During storage, various alterations occur in the properties of blood cells and the storage media, collectively referred to as “storage lesions,” which can compromise cell function and integrity (2, 4, 5). These changes include metabolic and biochemical shifts such as decreases in pH, glucose consumption, increases in lactate and potassium levels, and reductions in adenosine triphosphate (ATP) and 2,3-diphosphoglycerate (DPG). Morphological changes in RBCs during storage manifest as decreased deformability, echinocytosis, microparticle formation, and ultimately hemolysis, often attributed to oxidative injury (6, 7).

Oxidative injury in RBCs primarily arises from hemoglobin autoxidation, leading to the production of superoxide anions and reactive oxygen species (ROS). Antioxidant enzymes and molecules within RBCs play a crucial role in regulating these oxidative reactions; however, their dysfunction during storage makes RBCs more vulnerable to oxidative stress (8, 9). Additionally, leukocytes present in the blood bag contribute to storage lesions by releasing enzymes, cytokines, and oxygen radicals. Processed blood that has undergone leukocyte depletion exhibits lower levels of interleukins, bioactive proteins, oxidized hemoglobin, and erythrocyte ROS indices (10–14).

While previous studies in humans have sought to measure intraerythrocytic ROS during blood storage (15–18), no research has specifically focused on ROS in canine RBCs during this process. The current study aims to assess hematological and biochemical changes, quantify intraerythrocytic ROS and antioxidants, and explore the effects of pre-storage leukoreduction on storage lesions and ROS in canine RBCs throughout the storage period.

## 2 Materials and methods

### 2.1 Study design

This study involved an *in vitro* analysis of hematological and biochemical changes in canine packed red blood cells (pRBCs) during storage. The effect of leukocytes on various parameters was evaluated, with pRBC units categorized into leukoreduced pRBC (LR–pRBC) and non-leukoreduced pRBC (nLR–pRBC) units.

**Figure 1:**
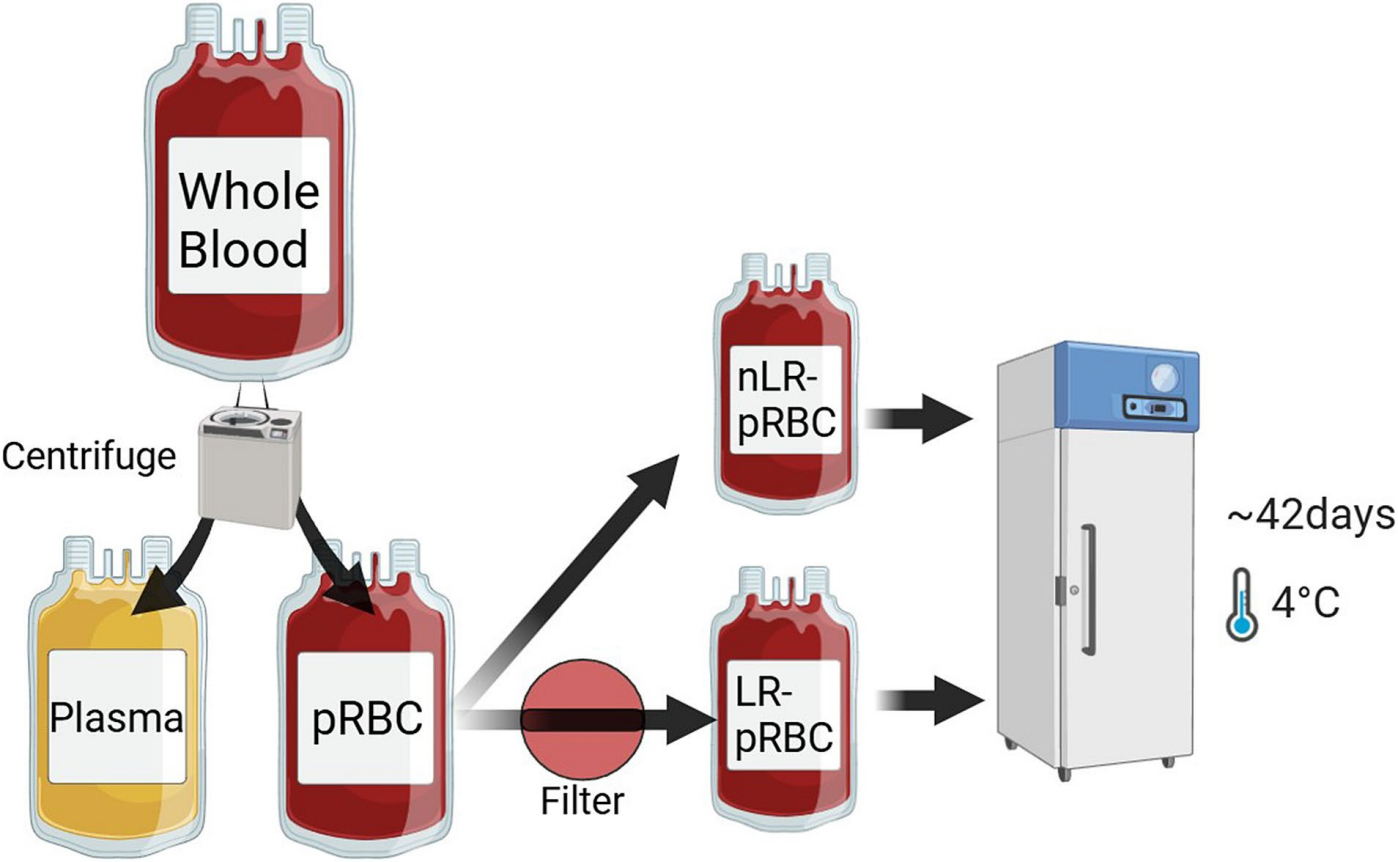
Blood separation and leukoreduction process of whole blood samples (*n* = 11). 11 whole blood samples were centrifuged and separated into pRBC and plasma. Half of the pRBCs were filtered with an in-line leukoreduction to obtain LR–pRBC units, while the other half remained unfiltered as nLR–pRBC units. All blood units were stored at 2–6°C for 6 weeks.

Eleven whole blood (WB) units were included, collected from healthy volunteer dogs using a quadruple bag collection system (14) integrated with a hard-type leukoreduction filter (In-line System, Changyoung Medical, Eumseong, Korea). The WB units were treated with citrate phosphate dextrose (CPD) solution as the anticoagulant and gently mixed in the primary bag. After stabilization at 4°C for at least 2 h, pRBCs were separated via centrifugation at 3,015 rpm for 8 min at 22°C (Component R6, Hanil Scientific Inc., Gimpo, Korea) and gently mixed with 88.9 mL of saline adenine glucose–mannitol (SAG–M) solution. The separated eleven packed red blood cell (pRBC) units were further divided into two aliquots. One half of the pRBCs was passed through a leukoreduction filter (RCM1, Haemonetics, MA, USA) and stored as LR–pRBC units, while the remaining half was stored unfiltered in a satellite bag as nLR–pRBC units. As a result of this process, eleven units each of LR-and nLR-pRBC were prepared and stored vertically in a blood refrigerator at 2–6°C for 6 weeks, with gentle agitation every 2 days during storage (Figure 1). Blood samples were collected from the pRBC units at 1, 7, 14, 21, 28, 35, and 42 days post-separation. The pRBC aliquot was collected sterilely, with half used for RBC lysates (by mixing with ice-cold deionized water) and the other half for hematological analysis, erythrocytic mean osmotic fragility (MOF) tests, and intraerythrocytic ROS measurement. RBC lysates were centrifuged and stored at −80°C for subsequent antioxidant analysis.

### 2.2 Analysis of storage lesions in LR– and nLR–pRBCs

To comprehensively evaluate storage lesions, we established evaluation parameters according to the following three analytical targets: Metabolic/biochemical changes: pH, potassium concentration, lactate concentration; Morphological changes: mean osmotic fragility (MOF), hemolysis (%); Oxidative injury parameters: ROS, superoxide Dismutase (SOD), catalase (CAT), glutathione peroxidase (GPx), and total antioxidant capacity (TAC).

Hematologic analysis included RBC count, hematocrit (Hct), total hemoglobin (Hb) concentration, and white blood cell (WBC) count, performed using a bench-top laboratory analyzer (ProCyte Dx hematology analyzer, IDEXX Laboratories, USA). The hemolysis percentage was calculated using the formula:


Hemolysis%=(100–Hct)×[supernatantHb(g/dL)/totalHb(g/dL)]

To evaluate deformability, RBC sphericity, and resistance to hemolysis, the erythrocyte MOF test was conducted as per previous studies (13, 19). The blood samples were incubated for 30 min in isotonic-to-hypotonic sodium chloride solutions (0.85, 0.80, 0.75, 0.70, 0.65, 0.60, 0.55, 0.50, 0.45, 0.40, 0.35, 0.30, 0.20, and 0.10% NaCl) in room temperature. After centrifuge at 1,500 rpm for 5 min, the separated supernatant was dispensed into a 96-well microplate (Stripwell Microplate, Corning Inc., USA), and optical densities (ODs) were measured at 540 nm using a spectrophotometer (Spectra Max M2, Molecular Devices, USA) and analytic software (SoftMax Pro, Molecular Devices, USA). MOF values correspond to the diluted solution concentration at which 50% hemolysis occurs, determined from the lysis curve. The percentage of hemolysis for each sample was calculated using the following formula:


$$\text{Hemolysis}\;\%=({\mathrm{OD}}_{\exp}-{\mathrm{OD}}_{0.1\%})\times 100$$

The OD_exp_ refers to the optical density of the test sample at a given NaCl concentration, while OD_0.1%_ represents the optical density measured in the completely hemolyzed sample (0.1% NaCl). To evaluate metabolic changes, Supernatant pH, lactate, and potassium concentrations were measured using a commercial laboratory analyzer (Nova pHOX Ultra analyzer, Nova Biomedical, USA), with detection limits of 6.5 for pH and 20.0 mmol/L for lactate concentration.

### 2.3 The intraerythrocytic ROS and antioxidants analysis

**Figure 2:**
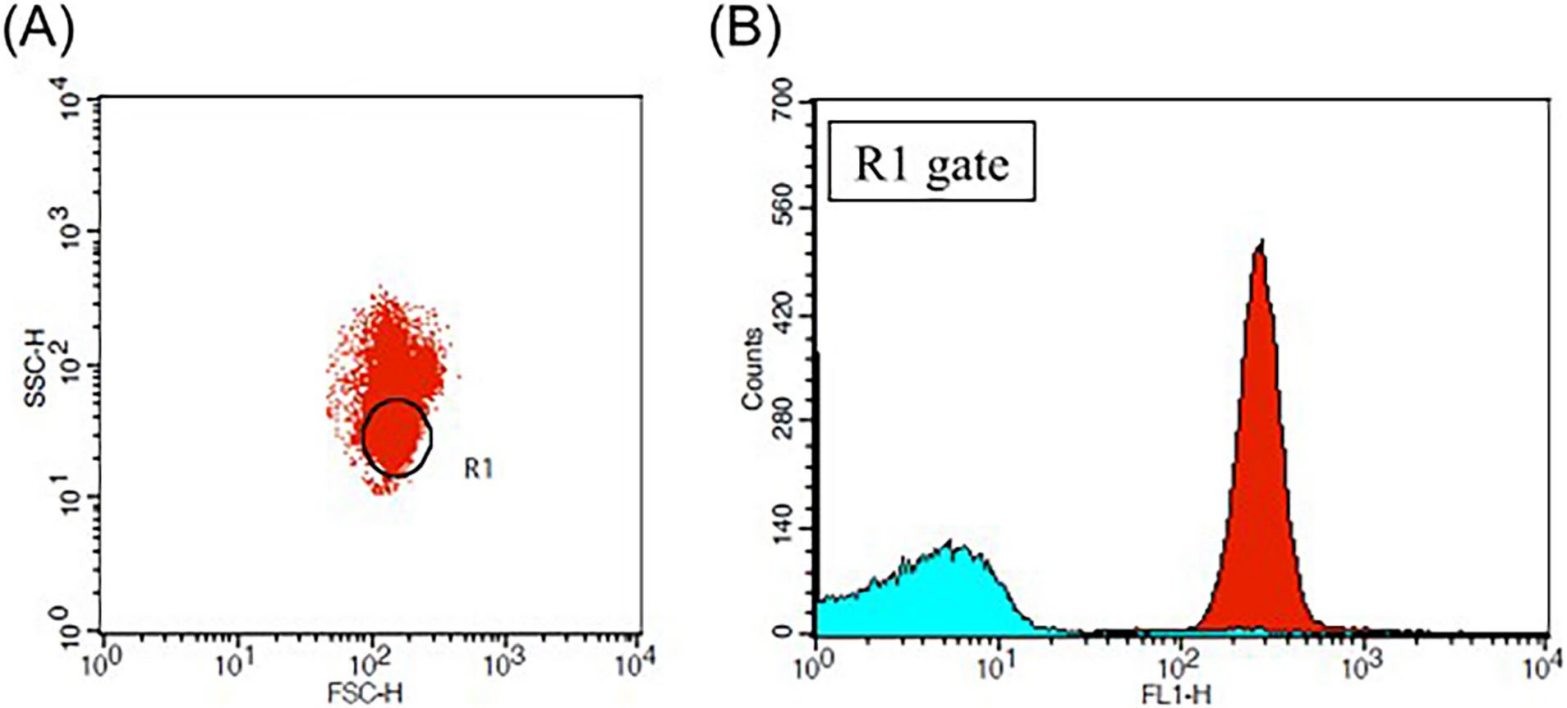
Flow cytometric analysis of pRBC treated with carboxy–H_2_DCFDA. **(A)** Two-parameter dot plot analyzing the log scale of forward scatter (FSC) and side scatter (SSC), with the R1 gate indicating the location of the red blood cell (RBC) population **(B)** Histogram plot of the FL–1 laser for the R1 gate, where the red plot corresponds to the blood sample treated with 40 μM of carboxy–H2DCFDA and the sky-blue plot represents the blood sample treated with DMSO as a negative control. The mean fluorescence intensity (MFI) of the FL–1 laser reflects the levels of intraerythrocytic reactive oxygen species (ROS).

Carboxy–H2DCFDA (Invitrogen, Waltham, MA, USA) was utilized to detect intraerythrocytic ROS. pRBCs in 1% PBSA were incubated with 40 μM carboxy–H2DCFDA at 37°C for 20 min and analyzed immediately by flow cytometry (FACS–Calibur, Becton–Dickinson, USA). A total of 50,000 events were collected at a flow rate of 1,000 per second using CellQuest Pro software (Becton–Dickinson, Franklin Lakes, NJ, USA). The RBC population’s location was confirmed with reference to previous studies (17, 18) (Figure 2). ROS reactivity was represented by histograms and the mean fluorescence intensity (MFI) of FL-1 fluorescence using commercial software (FlowJo Software, Becton–Dickinson, Franklin Lakes, NJ, USA).

To evaluate antioxidative capacity, SOD, CAT, GPx, and TAC were measured from stored RBC lysate samples using commercial colorimetric activity kits (SOD and CAT Colorimetric Activity Kits from Invitrogen, USA; GPx Assay Kit from Cayman Chemical, USA; Total Antioxidant Assay Kit from Sigma-Aldrich, USA). All procedures were conducted according to the manufacturer’s instructions. Microplate absorbance was measured using a spectrophotometer (Spectra Max M2, Molecular Devices, USA) and analyzed with software (SoftMax Pro, Molecular Devices, USA).

### 2.4 Statistical analysis

Statistical analysis was conducted using commercial software (IBM SPSS, version 25, Chicago, IL, USA). Analytic data were tested for normality using the Shapiro–Wilk test. Except for RBC parameter (which were not included in the present analysis), all variables failed to meet the assumption of normality and analyzed using non-parametric statistical methods. The Wilcoxon signed-rank test was employed to compare the LR and nLR groups at each storage time point. To evaluate temporal changes within each group across the storage period, the Friedman test was utilized. The paired t-test (parametric) or Mann–Whitney U test (non-parametric), along with two-way repeated measures ANOVA or Kruskal–Wallis non-parametric test, were employed to assess hematological differences between LR and non-LR groups and storage duration effects. *Post hoc* analysis utilized a Tukey test (parametric) or Bonferroni correction and Mann–Whitney U non-parametric test. The Kruskal–Wallis and Mann–Whitney U tests compared MOF test results, intraerythrocytic ROS MFI, and antioxidant concentrations between LR and non-LR groups, as well as storage period effects. Spearman’s rank correlation analysis was performed to analyze correlations between variables. Data are presented as mean ± standard deviation (SD). If results were below or exceeded detection limits, they were represented as the limit value. The significance level for statistical tests was set at *p* < 0.05.

## 3 Results

### 3.1 Storage lesions of LR-pRBC and nLR-pRBC units

**Figure 3:**
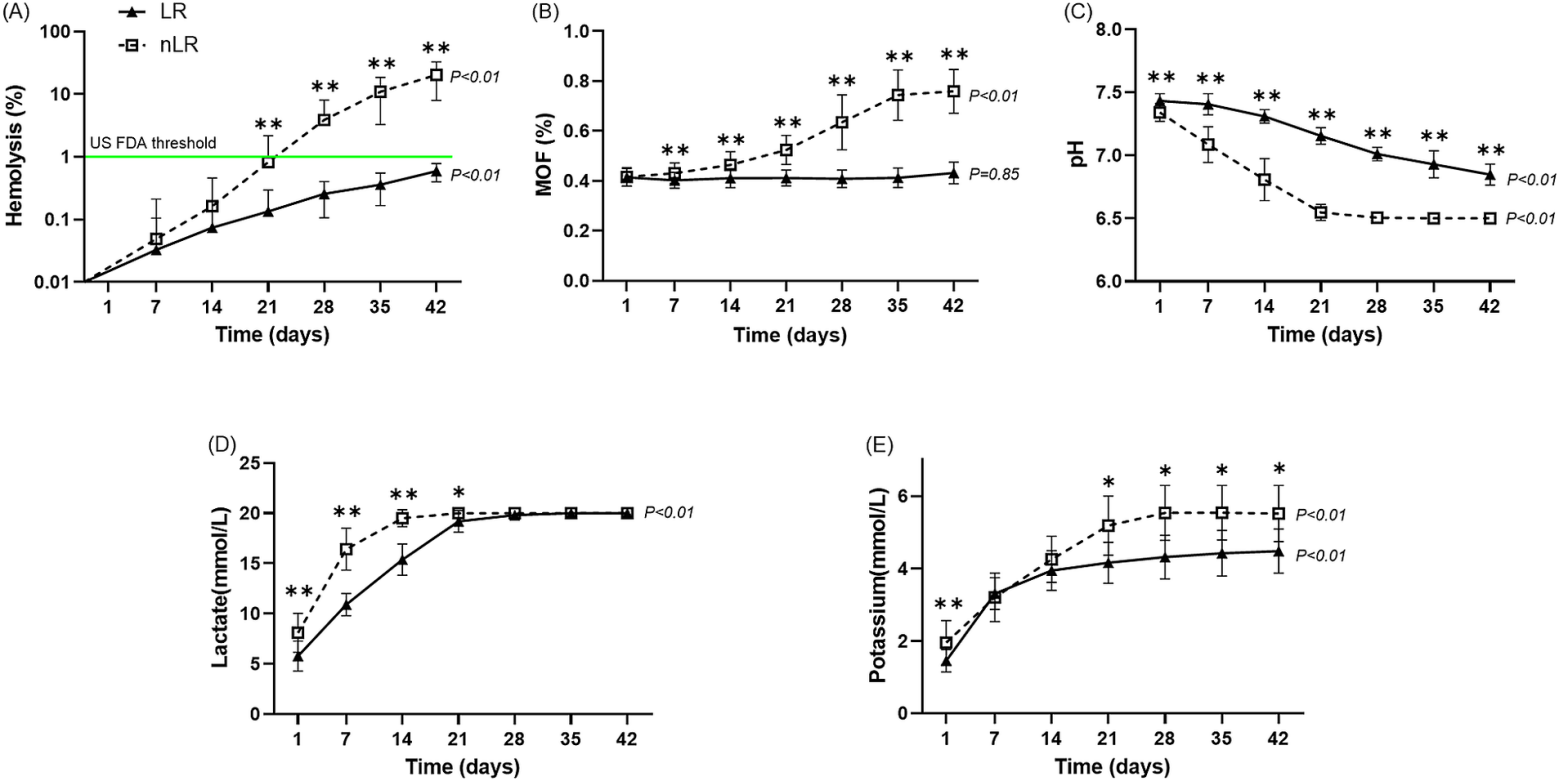
Storage lesions of the LR– and nLR–pRBC units (*n* = 11) during the storage period. The horizontal axis indicates the number of days of storage, while the vertical axis indicates the outcomes of hematological and biochemical analyses. The analytic results for the LR– and nLR–pRBC units are illustrated using a solid line and black-filled triangles and a dotted line and open squares, respectively. The results are presented as median and interquartile range (IQR). The *p*-value at the end of the graph represents statistical significance over the storage period. **(A)** Percentage of hemolysis; **(B)** mean osmotic fragility results; **(C)** pH level; **(D)** lactate concentration; and **(E)** potassium concentration. *p < 0.05 LR–pRBC vs. nLR–pRBC at each time point. **p < 0.01 LR–pRBC vs. nLR–pRBC at each time point.

The hemolysis percentage increased from day 1 to 42 in both groups, with a significant rise from day 21 to day 42 in the nLR-pRBC group (*p* < 0.01; Figure 3A). Although the hemolysis percentage also increased in the LR-pRBC group, it remained below 1%, which is the US FDA threshold for humans (20). Notably, the hemolysis percentage on day 21 significantly correlated with the WBC count on the first day of storage (*p* < 0.05) (Table 1).

**Table 1:** WBC count of LR– and nLR–pRBCs (*n* = 11) during storage periods.

Day(s)	Mean WBC count 10^3^/μL (SD)	
	LR	nLR
D1	0.003 (0.005)	22.948 (5.419)
D7	0.003 (0.006)	23.313 (6.888)
D14	0.005 (0.005)	20.414 (5.488)
D21	0.005 (0.007)	19.005 (4.190)
D28	0.004 (0.005)	16.143 (5.048)
D35	0.003 (0.005)	11.764 (7.164)
D42	0.006 (0.005)	8.995 (6.203)

The MOF values in the LR-pRBC group did not change significantly over the storage period (*p* = 0.44), whereas the nLR-pRBC group showed a significant increase during storage (*p* < 0.01). Notably, a significant difference between the two groups was first observed on day 7 of storage (*p* < 0.05), and this difference became more pronounced with prolonged storage (Figure 3B).

Significant differences in pH and lactate levels were observed between storage periods in both groups (*p* < 0.01), with more pronounced changes in the nLR-pRBC group (Figures 3C,D). Lactate concentration remained unchanged in both groups from day 21 to 42 due to the upper detection limit (20 mmol/L). The potassium concentration significantly increased with storage duration in both groups (*p* < 0.01; Figure 3E), with nLR-pRBC units showing significantly higher potassium levels on days 1 (*p* < 0.05), 21, 28, 35, and 42 (*p* < 0.01).

### 3.2 Intraerythrocytic ROS and antioxidant levels over the storage period

**Figure 4:**
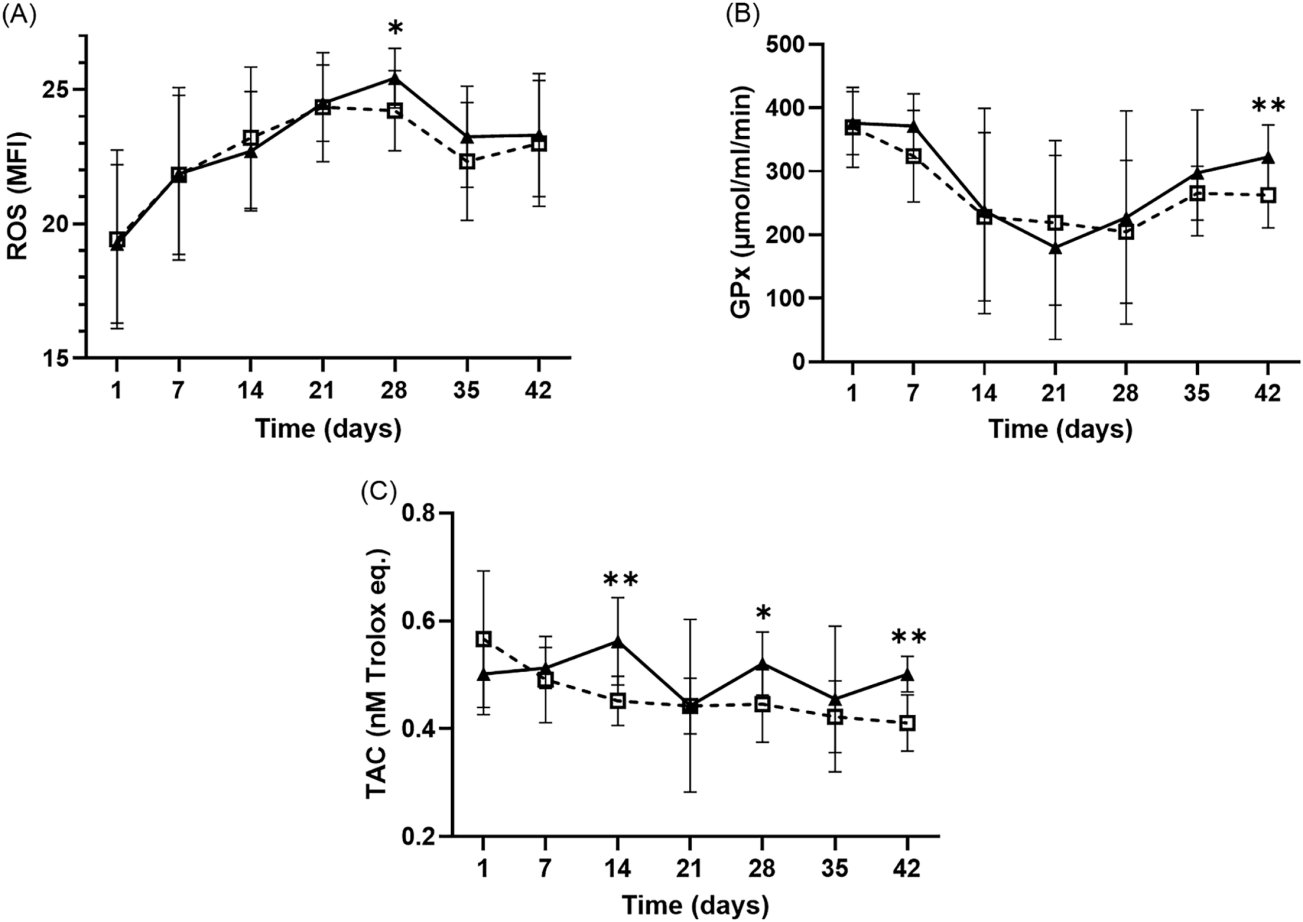
Intraerythrocytic ROS results **(A)** and antioxidant analysis of GPx **(B)** and TAC **(C)** during the storage period of LR– and nLR–pRBC units. The intraerythrocytic ROS and antioxidants (TAC and GPx) was measured in the LR–pRBCs (solid line, black–filled triangles) and nLR–pRBCs (dotted line, blank squares) groups. Sample size of ROS and TAC is 11, and that of GPx is 10. *p < 0.05 LR–pRBC vs. nLR–pRBC at each time point. **p < 0.01 LR–pRBC vs. nLR–pRBC at each time point.

Significant differences in intraerythrocytic ROS levels were observed across different storage durations in both groups (Figure 4A). The MFI gradually increased until day 28 and then decreased for both groups. Notably, the MFI of ROS in the LR-pRBC group was significantly higher than that in the nLR-pRBC group on day 28 (*p* < 0.05). Conversely, GPx activity decreased until day 21 and then increased until the end of the study, with significantly higher GPx activity in the LR-pRBC group than in the nLR-pRBC group on day 42 (*p* < 0.05; Figure 4B). The TAC concentration in the nLR-pRBC group significantly decreased over the storage period (*p* < 0.01; Figure 4C), whereas the TAC concentration was significantly higher in the LR-pRBC group on days 14 (*p* < 0.01), 28 (*p* < 0.05), and 42 (*p* < 0.01). Changes in SOD and CAT levels did not significantly differ between groups over the storage period (data not shown).

## 4 Discussion

The purpose of the present study was to evaluate hematological/biochemical storage lesions, the intraerythrocytic ROS using fluorochrome, and antioxidants concentration during blood storage periods, and confirm the correlation with leukoreduction. Throughout the storage periods, hemolysis, lactic acidosis, and potassium efflux increased in both groups, with these variations being more pronounced in the nLR–pRBC group, likely due to the presence of leukocytes. The intraerythrocytic ROS levels gradually increased in both groups until day 28, but no significant differences were observed between groups. GPx activity decreased steadily in both groups until day 21, while total antioxidant capacity (TAC) concentration declined only in the nLR–pRBC group. A significant correlation was found between the white blood cell (WBC) count on the first day and the hemolysis percentage on day 21 in the nLR–pRBC group. Additionally, the mean osmotic fragility (MOF) value was significantly higher after day 7 in the nLR–pRBC group compared to the LR–pRBC group, which is consistent with previous studies that found MOF to be higher in the nLR-pRBC group than in the LR-pRBC group on day 7 (13).

In the present study, the supernatant pH declined significantly in both pRBC groups over the storage period and was markedly lower in the nLR-pRBC group compared to the LR-pRBC group. In contrast, supernatant lactate concentrations increased significantly during storage in both groups and were consistently higher in the nLR-pRBC group than in the LR-pRBC group. The decrease in pH in stored blood units is attributable to the accumulation of lactate, a metabolic byproduct of the glycolytic pathway (3, 21). Because mature mammalian erythrocytes lack mitochondria, ATP generation required for maintaining cellular functionality and membrane integrity relies exclusively on anaerobic glycolysis (2, 3, 21). During storage, elevated lactate concentrations inhibit the glycolytic pathway through negative feedback mechanisms and lactic acidosis (3, 21), consequently reducing intracellular ATP levels. In a cold environment compared to room temperature, the glycolysis process and ATP synthesis in RBCs can be inhibited, and the lactaic acids produced by glycolysis and the resulting acidic environment can be alleviated (22). Since the cellular functions of RBCs that require ATP are also reduced in a cold environment, it is thought that cold storage may actually be beneficial for ATP synthesis in RBCs in the long term (21). Although ATP concentration was not assessed in the current study, a progressive decline is expected to occur throughout the storage period, likely contributing to the deterioration of RBC structural and functional properties, as shown in the previous study (13). Leukoreduction attenuated the degree of acidosis and hyperlactatemia both in the current investigation and in prior reports (13), potentially due to decreased glucose consumption by residual leukocytes (10, 23). Since the upper limit of the lactate measuring equipment used in this experiment is 20 mmol/L, it is believed that the lactate concentration in the nLR group is actually significantly higher than that in the LR group even after the 14th day. The reduction in the difference between groups is presumed to be a result of the decreased glucose consumption by residual leukocytes, as mentioned earlier. It is thought that leukocytes also decrease in number over the storage period because the synthesis of ATP decreases as glucose is consumed within the stored blood, leading to apoptosis.

Moreover, the supernatant potassium concentration increased significantly throughout the storage duration in both experimental groups. Notably, potassium levels were significantly higher in the nLR-pRBC group than the LR-pRBC group from the initial day of storage and persisted through the later stages. This trend is consistent with previous canine studies (13, 23, 24). However, no significant differences were observed between leukoreduced and non-leukoreduced units in one report (24). Under physiological conditions, RBC membranes exhibit limited permeability to monovalent cations, a balance maintained by membrane-bound Na^+^/K^+^-ATPase activity. However, during hypothermic storage, diminished ATP availability compromises Na^+^/K^+^-ATPase function, resulting in elevated extracellular potassium and increased intracellular sodium concentrations (21, 25). Typically, canine erythrocytes exhibit low intracellular potassium levels due to minimal Na^+^/K^+^-ATPase activity (3). Additionally, the composition of additive solutions influences potassium efflux, with CPDA-1 producing lower supernatant potassium levels compared to Adsol and Nutricel solutions due to their differing ionic constituents (23). Oxidative stress has been shown to reduce the activity of membrane-associated enzymes, including Na^+^/K^+^-ATPase, suggesting that intraerythrocytic reactive oxygen species (ROS) may further facilitate potassium leakage into the storage medium (26). The presence of leukocytes in the blood bag promotes glucose consumption, thereby impairing ATP synthesis in RBCs and compromising Na^+^/K^+^-ATPase activity. In addition, ROS and cytokines released by leukocytes may increase erythrocyte membrane permeability. For these reasons, supernatant potassium levels were significantly higher in the nLR-pRBC groups than in the LR-pRBC in this study. The rate of potassium accumulation appeared less pronounced in the later stages of storage, potentially due to a reduced transmembrane cation gradient (25).

Despite the absence of leukocytes, storage lesions, including increased hemolysis percentage, were observed in the LR–pRBC group. While the influence of unfiltered interleukins and ATP degradation cannot be dismissed, it seems that oxidative damage to intracellular proteins and lipids caused by intraerythrocytic ROS, which was resulting from hemoglobin autoxidation during storage, may induce these lesions. The increased intraerythrocytic ROS levels and the corresponding decrease in GPx activity reflect oxidative injury to RBCs during the storage period, a finding corroborated by previous studies (10, 27, 28). No significant differences in ROS levels were noted between the two groups. Although the exact mechanisms remain unclear, it is plausible that unfiltered interleukins in blood bags may affect ROS production in the LR–pRBC group even after leukoreduction (11, 12, 14).

The trend of ROS-dependent fluorescence intensity in RBCs was similar to the results of two human studies (10, 27), where fluorescence intensities generally decreased during the latter part of storage. This decline in fluorescence was associated with the leakage of fluorochrome from RBCs due to hemolysis, micro-vesiculation, or reduced esterase activity during storage (6, 7, 10). Conventionally, H2DCFDA can penetrate the lipophilic cellular membrane and localize in the cell’s aqueous compartment (29, 30). However, a recent study suggested that H2DCFDA, H2DCF, and DCF may also reside within a liposomal bilayer of the cell membrane (31). It is believed that during hemolysis or micro-vesiculation, oxidized hemoglobin and ROS—products of hemoglobin autoxidation—leak out of the cells, leading to decreased detectable fluorescence intensity in later storage periods. In this study, the timing of the decrease in MFI coincided with increases in hemolysis percentage and MOF results. Notably, the significantly higher ROS levels observed on day 28 in the LR–pRBC group compared to the nLR–pRBC group were associated with the relatively earlier decrease in intraerythrocytic ROS in the nLR–pRBC group (Table 1).

Previous studies have shown that TAC can serve as an antioxidant indicator in canine blood (32–34). In this study, the TAC concentration decreased only in the nLR–pRBC group, with some time points showing much higher levels in the LR–pRBC group. Although further research is needed regarding the changes in the concentration of TAC, it is determined that the overall oxidative damage of RBC was more severe in the nLR-RBC group. Possible causes may include the residual leukocytes promoting the generation of ROS and the degradation of antioxidants present in the blood (such as glutathione and vitamin C) over time, which may have accelerated oxidative damage.

When applying the US FDA criteria (20), on the last day of storage (day 42), every unit of LR–pRBC met the hemolysis criteria, while only one unit in the nLR–pRBC group fulfilled the criterion, with all others failing to meet the requirement. Additionally, other storage lesions were observed to be much more severe in the nLR–pRBC group. Based on these study results, it is suggested that leukoreduction is recommended to enhance blood storage stability and preemptively remove leukocyte-derived pathogens.

This study had several limitations. First, other factors might have influenced oxidative damage and storage lesions, such as leukocyte-derived ROS, interleukins, and ATP concentrations during pRBC storage. A recent study shows that during storage, ATP and antioxidants were de-creased with elevating free short and medium-chain fatty acids and leukoreduction alleviated these storage lesions (35). Second, the small sample size may introduce statistical bias or variability. Therefore, further studies are needed to control for leukocyte effects on storage lesions, intraerythrocytic ROS, and antioxidants. Recent clinical study results have been reported, indicating that there is no correlation between leukoreduction and the occurrence of transfusion reactions in dogs who have actually received transfusions (36–38). However, through this experiment, it was confirmed that various storage lesions occur when leukoreduction is not performed. While this may have little relation to transfusion reactions that directly worsen the dog’s condition, it is believed to potentially hinder the effectiveness of transfusions, which are an important therapeutic method in veterinary medicine.

In conclusion, storage lesions such as hemolysis, lactic acidosis, and potassium efflux occur during blood storage. It has been confirmed that leukocytes in nLR–pRBC cause more severe storage lesions compared to LR–pRBC. Regardless of leukocyte presence, intraerythrocytic ROS levels increase during blood storage due to hemoglobin autoxidation. Elevated ROS appears to induce storage lesions, which include decreased antioxidants, hemolysis, osmotic fragility, and cation leakage in LR–pRBC. The ROS levels decline in the later stages of storage as a result of hemolysis, membrane vesiculation, and reduced esterase activity. To meet the criteria for pRBC storage and mitigate storage lesions, pre-storage leukoreduction is recommended. Further studies are necessary to explore the effects of antioxidant systems on intraerythrocytic ROS and storage lesions.

## Data Availability

The original contributions presented in the study are included in the article/Supplementary material, further inquiries can be directed to the corresponding author.
