# Supplementary material for: Understanding the biochemical impact of leukoreduction on canine pRBC storage: a focus on reactive oxygen species and storage lesions
Source: Front Vet Sci. 2025 Sep 4;12:1563532. doi: 10.3389/fvets.2025.1563532 (PMC12445046; doi:10.3389/fvets.2025.1563532)
Supplement: Supplementary file 1 [file Table_1.docx]

Supplementary 1.

| **Parameter** | **Storage Day** | **LR Mean (SD)** | **NLR Mean (SD)** | **p-value** |
| --- | --- | --- | --- | --- |
| Hemolysis (%) | 1 | 0.000 (0.000) | 0.000 (0.000) | ns |
|  | 7 | 0.033 (0.073) | 0.049 (0.162) | *0.651* |
|  | 14 | 0.074 (0.121) | 0.163 (0.295) | *0.637* |
|  | 21 | 0.134 (0.160) | 0.821 (1.349) | *0.051* |
|  | 28 | 0.255 (0.149) | 3.861 (4.199) | ***0.001*** |
|  | 35 | 0.360 (0.194) | 10.916 (7.655) | ***0.000*** |
|  | 42 | 0.590 (0.191) | 20.387 (12.393) | ***0.000*** |
| MOF (%) | 1 | 0.415 (0.035) | 0.416 (0.037) | *0.683* |
|  | 7 | 0.403 (0.032) | 0.431 (0.041) | ***0.034*** |
|  | 14 | 0.411 (0.039) | 0.465 (0.052) | ***0.003*** |
|  | 21 | 0.412 (0.032) | 0.525 (0.059) | ***0.000*** |
|  | 28 | 0.409 (0.036) | 0.635 (0.110) | ***0.000*** |
|  | 35 | 0.413 (0.040) | 0.745 (0.101) | ***0.000*** |
|  | 42 | 0.432 (0.044) | 0.760 (0.088) | ***0.000*** |
| pH | 1 | 7.433 (0.058) | 7.340 (0.072) | ***0.004*** |
|  | 7 | 7.405 (0.085) | 7.085 (0.142) | ***0.000*** |
|  | 14 | 7.309 (0.054) | 6.807 (0.167) | ***0.000*** |
|  | 21 | 7.155 (0.065) | 6.547 (0.063) | ***0.000*** |
|  | 28 | 7.012 (0.055) | 6.505 (0.014) | ***0.000*** |
|  | 35 | 6.928 (0.108) | 6.500 (0.000) | ***0.000*** |
|  | 42 | 6.847 (0.083) | 6.500 (0.000) | ***0.000*** |
| Lactate (mmol/L) | 1 | 5.782 (1.505) | 8.100 (1.950) | ***0.004*** |
|  | 7 | 10.909 (1.107) | 16.427 (2.076) | ***0.000*** |
|  | 14 | 15.391 (1.570) | 19.518 (0.855) | ***0.000*** |
|  | 21 | 19.200 (1.091) | 20.000 (0.000) | ***0.007*** |
|  | 28 | 19.827 (0.326) | 20.000 (0.000) | *0.079* |
|  | 35 | 20.000 (0.000) | 20.000 (0.000) | ns |
|  | 42 | 20.000 (0.000) | 20.000 (0.000) | ns |
| Potassium (mmol/L) | 1 | 1.449 (0.312) | 1.951 (0.605) | *0.053* |
|  | 7 | 3.303 (0.438) | 3.202 (0.675) | *0.312* |
|  | 14 | 3.943 (0.548) | 4.249 (0.636) | ***0.015*** |
|  | 21 | 4.155 (0.567) | 5.185 (0.819) | ***0.006*** |
|  | 28 | 4.315 (0.604) | 5.532 (0.760) | ***0.008*** |
|  | 35 | 4.421 (0.631) | 5.537 (0.756) | ***0.015*** |
|  | 42 | 4.477 (0.606) | 5.515 (0.776) | *0.603* |
| ROS (MFI) | 1 | 19.251 (2.955) | 19.422 (3.321) | *0.876* |
|  | 7 | 21.864 (3.216) | 21.822 (2.961) | *0.972* |
|  | 14 | 22.705 (2.225) | 23.202 (2.634) | *0.795* |
|  | 21 | 24.496 (1.424) | 24.350 (2.034) | *0.735* |
|  | 28 | 25.427 (1.117) | 24.215 (1.493) | ***0.046*** |
|  | 35 | 23.242 (1.882) | 22.324 (2.191) | *0.359* |
|  | 42 | 23.303 (2.292) | 22.992 (2.340) | *0.931* |
| GPx (μmol/ml/min) | 1 | 376.130 (49.685) | 369.360 (63.022) | *0.793* |
|  | 7 | 371.364 (50.866) | 323.835 (72.072) | *0.104* |
|  | 14 | 237.764 (161.849) | 228.601 (132.651) | *0.308* |
|  | 21 | 180.077 (145.052) | 218.891 (129.816) | *0.970* |
|  | 28 | 227.133 (167.889) | 204.967 (112.776) | *0.345* |
|  | 35 | 297.673 (99.048) | 265.521 (42.682) | *0.064* |
|  | 42 | 322.800 (50.331) | 262.754 (51.821) | ***0.017*** |
| TAC (nM Trolox eq.) | 1 | 0.502 (0.076) | 0.566 (0.126) | *0.161* |
|  | 7 | 0.513 (0.038) | 0.491 (0.080) | *0.432* |
|  | 14 | 0.562 (0.081) | 0.452 (0.046) | ***0.001*** |
|  | 21 | 0.443 (0.160) | 0.442 (0.051) | *0.994* |
|  | 28 | 0.521 (0.059) | 0.446 (0.070) | ***0.014*** |
|  | 35 | 0.455 (0.135) | 0.422 (0.066) | *0.076* |
|  | 42 | 0.501 (0.033) | 0.411 (0.052) | ***0.000*** |
